# Supplementary material for: Machine Learning Accelerates Crystallization for Structure Determination
Source: Angew Chem Int Ed Engl. 2026 May 4;65(25):e1218503. doi: 10.1002/anie.1218503 (PMC13266958; doi:10.1002/anie.1218503)

checkCIF/PLATON report

Structure factors have been supplied for datablock(s) lcz016

THIS REPORT IS FOR GUIDANCE ONLY. IF USED AS PART OF A REVIEW PROCEDURE FOR PUBLICATION, IT SHOULD NOT REPLACE THE EXPERTISE OF AN EXPERIENCED CRYSTALLOGRAPHIC REFEREE.

No syntax errors found. CIF dictionary Interpreting this report

Datablock: lcz016

Bond precision:	C-C = 0.0051 A		Wavelength=1.54184
Cell:	a=12.7346(1)	b=11.2852(1)	c=22.5392(1)
	alpha=90	beta=95.545(1)	gamma=90
Temperature:	100 K		

	Calculated	Reported
Volume	3224.01(4)	3224.01(4)
Space group	P 21/n	P 1 21/n 1
Hall group	-P 2yn	-P 2yn
Moiety formula	C15 H3 Ag3 F18 N6, C9 H10 O3	C15 H3 Ag3 F18 N6, C9 H10 O3
Sum formula	C24 H13 Ag3 F18 N6 O3	C24 H13 Ag3 F18 N6 O3
Mr	1099.01	1099.01
Dx,g cm-3	2.264	2.264
Z	4	4
Mu (mm-1)	15.841	15.841
F000	2104.0	2104.0
F000'	2114.37	
h,k,lmax	16,14,28	16,14,28
Nref	6905	6658
Tmin,Tmax	0.187,0.205	0.176,1.000
Tmin'	0.034	

Correction method= # Reported T Limits: Tmin=0.176 Tmax=1.000
AbsCorr = MULTI-SCAN

```
Data completeness= 0.964          Theta(max)= 77.976

                                                wR2(reflections)=
R(reflections)= 0.0364( 6255)                   0.1024( 6658)

S = 1.068                 Npar= 490
```

The following ALERTS were generated. Each ALERT has the format
test-name_ALERT_alert-type_alert-level.
Click on the hyperlinks for more details of the test.

Alert level C

```
PLAT911_ALERT_3_C Missing FCF Refl Between Thmin & STh/L=    0.600          4 Report
               -1  3  1,  -6  0  8,   5 11 12, -10  0 22,
```

Alert level G

```
PLAT007_ALERT_5_G Number of Unrefined Donor-H Atoms .............           1 Report
              H00D
PLAT142_ALERT_4_G s.u. on b - Axis Small or Missing .............     0.00010 Ang.
PLAT143_ALERT_4_G s.u. on c - Axis Small or Missing .............     0.00010 Ang.
PLAT153_ALERT_1_G The s.u.'s on the Cell Axes   are Equal ..(Note)     0.0001 Ang.
PLAT242_ALERT_2_G Low    'MainMol' Ueq as Compared to Neighbors of       C014 Check
PLAT242_ALERT_2_G Low    'MainMol' Ueq as Compared to Neighbors of       C018 Check
PLAT242_ALERT_2_G Low    'MainMol' Ueq as Compared to Neighbors of       C01C Check
PLAT242_ALERT_2_G Low    'MainMol' Ueq as Compared to Neighbors of       C01D Check
PLAT242_ALERT_2_G Low    'MainMol' Ueq as Compared to Neighbors of       C01H Check
PLAT434_ALERT_2_G Short Inter HL..HL Contact F00Q     ..F00U     .       2.82 Ang.
                                                  1+x,y,z  =      1_655 Check
PLAT720_ALERT_4_G Number of Unusual/Non-Standard Labels .........          67 Note
              Ag01    Ag02    Ag03    F004    F005    F006    F007    F008
              F009    F00A    F00B    F00C    O00D    H00D    F00E    F00F
              F00G    O00H    O00I    F00J    N00K    F00L    N00M    N00N
              N00O    F00P    F00Q    F00R    N00S    N00T    F00U    C00V
              H00V    C00W    C00X    C00Y    C00Z    C010    C011    H011
              C012    C013    C014    C015    H015    C016    H016    C017
              H017    C018    C019    C01A    C01B    C01C    C01D    C01E
              H01E    C01F    C01G    H01A    H01B    H01C    C01H    C01I
              H01D    H01F    H01G
PLAT883_ALERT_1_G Absent Datum for _atom_sites_solution_primary ..     Please Do !
PLAT910_ALERT_3_G Missing FCF Reflection(s) Below Theta(Min)[Deg]=       4.17 Note
               -1  0  1,   0  0  2,
PLAT912_ALERT_4_G Missing # of FCF Reflections Above STh/L=  0.600        241 Note
PLAT913_ALERT_3_G Missing # of Very Strong Reflections in FCF ....          1 Note
               -1  3  1,
PLAT941_ALERT_3_G Average HKL Measurement Multiplicity ..........         3.1 Low
PLAT969_ALERT_5_G The 'Henn et al.' R-Factor-gap value ..........       3.070 Note
              Predicted wR2: Based on SigI**2  3.33 or SHELX Weight  9.59
PLAT978_ALERT_2_G Number C-C Bonds with Positive Residual Density.          0 Info
```

---

0 **ALERT level A** = Most likely a serious problem - resolve or explain
0 **ALERT level B** = A potentially serious problem, consider carefully
1 **ALERT level C** = Check. Ensure it is not caused by an omission or oversight

```
 18 ALERT level G = General information/check it is not something unexpected

  2 ALERT type 1 CIF construction/syntax error, inconsistent or missing data
  7 ALERT type 2 Indicator that the structure model may be wrong or deficient
  4 ALERT type 3 Indicator that the structure quality may be low
  4 ALERT type 4 Improvement, methodology, query or suggestion
  2 ALERT type 5 Informative message, check
```

Validation response form

Please find below a validation response form (VRF) that can be filled in and pasted into your CIF.

```
# start Validation Reply Form
_vrf_PLAT911_lcz016
;
PROBLEM: Missing FCF Refl Between Thmin & STh/L=    0.600          4 Report
RESPONSE: ...
;
# end Validation Reply Form
```

It is advisable to attempt to resolve as many as possible of the alerts in all categories. Often the minor alerts point to easily fixed oversights, errors and omissions in your CIF or refinement strategy, so attention to these fine details can be worthwhile. It is up to the individual to critically assess their own results and, if necessary, seek expert advice.

PLATON version of 17/09/2025; check.def file version of 17/09/2025

---

duplicate check

No duplication found

Datablock lcz016 - ellipsoid plot

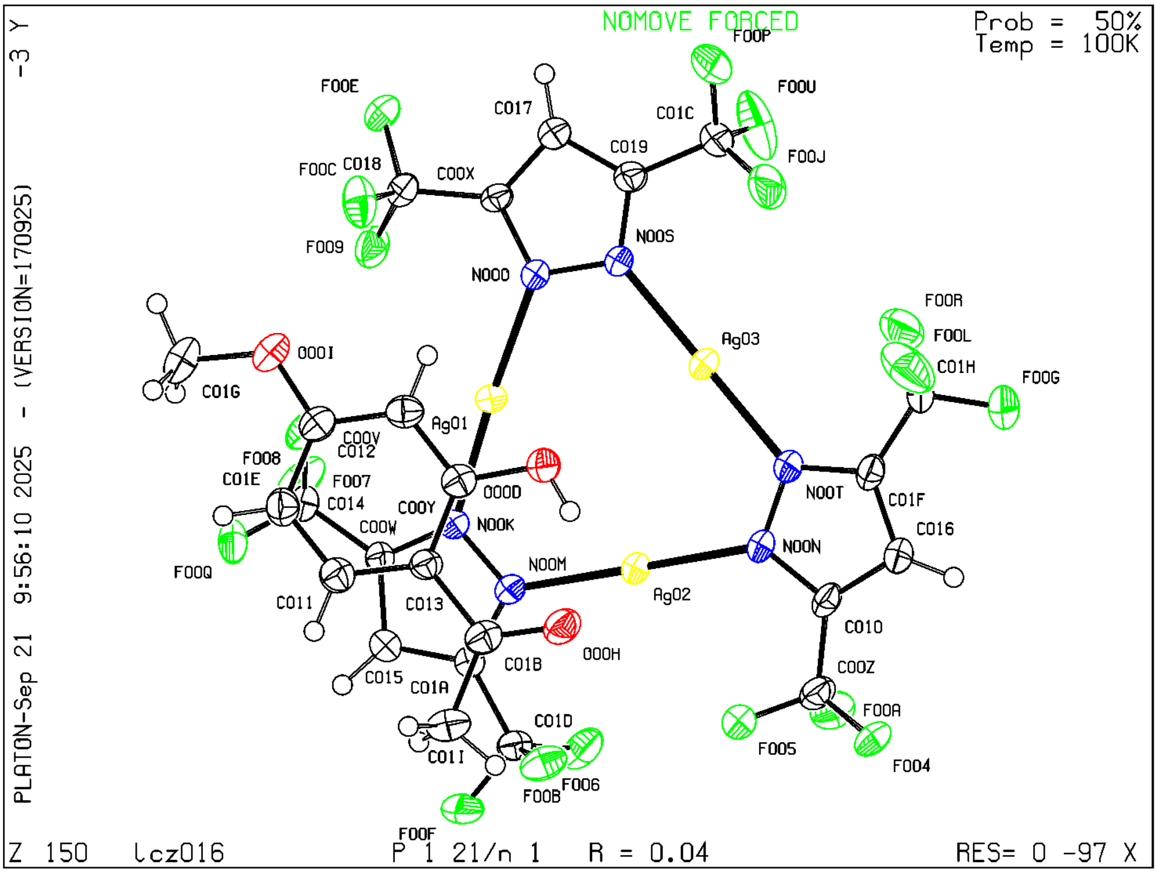

Supplement: Supplementary file 2 — Supporting File 2: anie72462‐sup‐0002‐DataFile.zip. [file ANIE-65-e1218503-s002.zip › supplementary_materials_crystallographic_data submit.zip!/cif_and_checkcif(1-114)/Ag3Pz3_16/Ag3Pz3_16_checkcif.pdf]
